# Multi-mode absorption spectroscopy using a quantum cascade laser for simultaneous detection of NO and H_2_O

**DOI:** 10.1007/s00340-016-6499-4

**Published:** 2016-08-04

**Authors:** S. O’Hagan, T. Pinto, P. Ewart, G. A. D. Ritchie

**Affiliations:** 1 Clarendon Laboratory, Department of Physics, Oxford University, Parks Road, Oxford, OX1 3PU UK; 2 Department of Chemistry, Physical and Theoretical Chemistry Laboratory, Oxford University, South Parks Road, Oxford, OX1 3QZ UK

**Keywords:** Absorption Line, Longitudinal Mode, Quantum Cascade Laser, Frequency Comb, Instrument Function

## Abstract

Detection of multiple transitions in NO and H_2_O using multi-mode absorption spectroscopy, MUMAS, with a quantum cascade laser, QCL, operating at 5.3 μm at scan rates up to 10 kHz is reported. The linewidth of longitudinal modes of the QCL is derived from pressure-dependent fits to experimental MUMAS data. Variations in the spectral structure of the broadband, multi-mode, output of the commercially available QCL employed are analysed to provide accurate fits of modelled MUMAS signatures to the experimental data.

## Introduction

Laser spectroscopy of gas phase species is most often conducted using single-mode lasers. Tunable diode laser absorption spectroscopy, TDLAS, using such lasers is a powerful and widely used technique for gas sensing applications including environmental monitoring, chemical analysis, plasma spectroscopy and combustion diagnostics [1–3]. The use of wavelengths in the spectral range 4–10 μm is advantageous since many important molecular species exhibit strong well-resolved absorption features in this range. Extension of laser-based methods to this region is therefore a desirable aim, and it has become more accessible by the use of commercially available quantum cascade lasers, QCLs. The high resolution available using single-mode devices provides high sensitivity with minimum detection limits in the range of ppbv in some applications. The limited range of mode-hop-free, MHF, tuning of most single-mode lasers, however, often restricts TDLAS methods to detection of a single species unless, fortuitously, two species have transitions lying within the MHF range. Detection of more than one species using TDLAS usually involves multiplexing several lasers and detector systems [4]. Widely tunable external cavity diode lasers, emitting narrow line or single-mode outputs, have recently been demonstrated in the 4–10 μm mid-infrared range based on quantum cascade devices but the relatively slow tuning rates limit application in rapidly changing environments [5]. A variety of broadband spectroscopy methods have been developed that allow detection of multiple species over a wide spectral range but not all are available in the mid-IR. These include use of multi-section diodes, broadband or wavelength agile light sources and correlation spectroscopic methods [6–12]. The wide bandwidth “frequency combs” available from mode-locked lasers has been exploited for broadband spectroscopy but usually involve complex laser systems and high resolution dispersion optics [13, 14]. Extension of comb-based spectroscopy to the mid-infrared beyond 4 μm is becoming possible with the development of frequency combs based on QCL devices. A comb of width 100 cm^−1^ has recently been demonstrated at 7 μm [15]. Heterodyne methods, with potential for multi-species sensing, have also been demonstrated to allow detection in the RF region at the cost of some additional complexity and restriction in width of the spectrum covered [16].

The technique of multi-mode absorption spectroscopy, MUMAS, addresses some of the limitations of TDLAS, specifically by providing wide spectral coverage whilst retaining high spectral resolution [17, 18]. The ability to simultaneously record multiple molecular transitions, and so detect multiple species, is achieved at the cost of higher minimum detection limits than provided by TDLAS methods using single-mode lasers. Nonetheless, applications that do not require the ultimate in detection sensitivity, such as in chemical reaction or industrial process monitoring or where target species are present in concentrations in the range of 10 ppmv or above, can benefit from the multi-species sensing capability and rapid response time available from MUMAS. Multi-species detection by MUMAS has been demonstrated in the near- and mid-infrared using a custom-built Er/Yb/glass microlaser at 1.5 μm, difference frequency generation, DFG, and, more recently, an interband cascade laser, ICL, in the 3–4 μm range [19–23]. Detected species in mixtures include, CO, CO_2_, CH_4_, C_2_H_2_, H_2_CO, NH_3_, N_2_O and H_2_O. Extension of MUMAS to wavelengths between 4 and 10 μm would have the advantage of exploiting the strong absorption lines in this region, increasing the number of molecular species that could be detected by the technique. It will be of further advantage if the technique can be implemented using commercially available QCL devices so that potential users will not be required to construct custom lasers or use relatively complex DFG systems.

In this paper, we report the first demonstration of MUMAS using a QCL and the first application of MUMAS to detect NO. In addition, data are obtained clearly distinguishing the presence of H_2_O in laboratory air showing the multi-species detection capability of the technique. We also show that the spectral width of the longitudinal modes of the multi-mode output of the QCL can be determined using measurements of pressure broadening of the MUMAS signatures. In addition, we present data obtained at laser scan rates of 10 kHz demonstrating the potential for short measurement times using QCLs for MUMAS. Significantly, the experiments were conducted using a commercially available multi-mode QCL whose emission spectrum was highly irregular and variable. Nonetheless, we demonstrate that, even with an irregular and spectrally structured multi-mode envelope, which also varied during the spectral scanning, all the features of experimentally recorded MUMAS signatures could be modelled with sufficient accuracy to achieve good fits to the data. These results demonstrate that commercially available multi-mode lasers can be used for MUMAS in spite of the far from optimal spectral characteristics of their output.

In what follows we present, firstly, a brief outline of the MUMAS technique and a description of the apparatus and the QCL employed. Attention is drawn to the important parameters of the laser that affect the form of the MUMAS signal and how they are measured to provide input data for the modelling process. Experimental results are then presented showing MUMAS data from samples of pure NO and also from NO in the presence of laboratory air containing water vapour. A detailed analysis is then presented of the effects on MUMAS signatures arising from variation, during the frequency scan, of the spectral envelope of the multi-mode spectrum emitted by the QCL. Finally, data obtained at scan rates up to 10 kHz are then presented to illustrate the potential for achieving short measurement times.

## Multimode absorption spectroscopy, MUMAS with a quantum cascade laser, QCL

### MUMAS procedure

Details of the principles and practice of MUMAS have been presented in previous publications and so are outlined only briefly here for convenience [17, 18]. The technique uses a conventional absorption spectroscopy approach in which the ratio of transmitted to incident intensity of a laser beam is measured for light propagating through a sample of gas contained in a pressure-controlled cell. In the case of MUMAS, all the longitudinal modes of the laser are scanned, typically across the frequency interval separating adjacent modes, $\Delta \nu_{\text{mode}}$. In this way, the whole range of the spectrum covered by the broadband output of the laser is interrogated. Each mode contains a certain proportion of the total power in the incident beam—the mode proportional power, $P_{\text{m}}$. If, during the scanning of the modes, an individual mode comes into resonance with an absorption transition in the gas, the total transmitted power is reduced by an amount that depends on the mode proportional power of that mode and the degree of absorption it experiences. A record of the variation of transmittance during the scan of the laser modes across the inter-mode spacing, $\Delta \nu_{\text{mode}}$, constitutes the MUMAS signature. Key parameters of the laser, apart from the inter-mode spacing, $\Delta \nu_{\text{mode}}$, are the width and shape of the laser spectrum or mode envelope, $\Delta \nu_{\text{band}}$, its location in frequency space, i.e. the frequency of the individual modes, $\nu_{\text{m}}$, and the individual mode linewidth, $\Delta \nu_{\text{width}}$. If each of these parameters is known, or can be measured, then the variation of the total transmitted power can be calculated, i.e. modelled, provided also that the locations and strengths of all molecular absorption features lying within $\Delta \nu_{\text{band}}$ are known. In most cases, spectral absorption data, line position and strength, are available from a suitable spectral database [24]. Other relevant parameters are the temperature and pressure in the gas cell that determine the Doppler and pressure broadening of the spectral lines.

### Experimental parameters

**Fig. 1:**
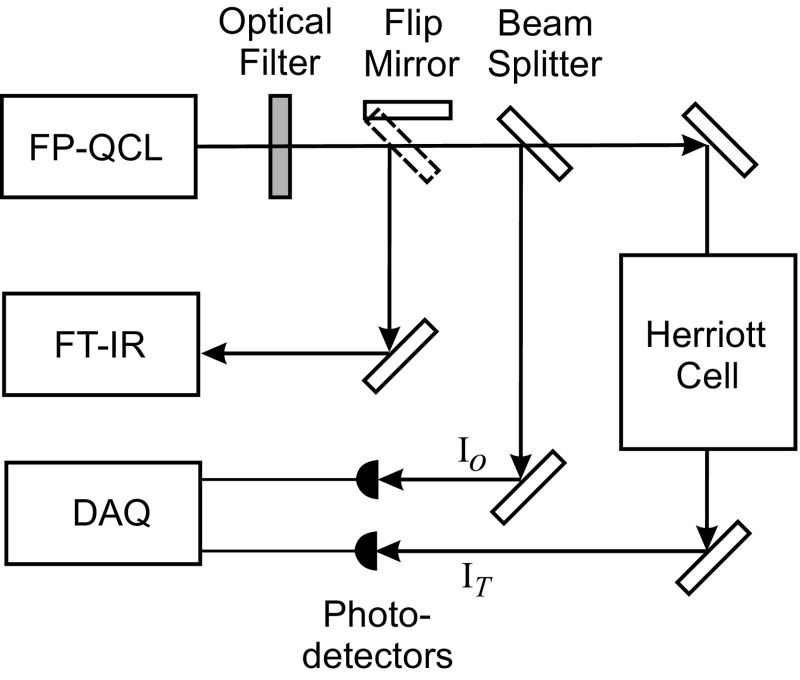
Schematic of the experimental layout. The spectrum of the output of the Fabry–Perot QCL is conditioned by the optical (interference) filter prior to being measured by the FTIR instrument. The flip mirror is removed during the MUMAS experiment in which the beam splitter reflects a small fraction of the beam to the upper photodetector to measure incident intensity, *I*_o_, and the remainder passes through the multi-pass Herriott cell containing the absorbing gas to the lower photodetector to measure the transmitted intensity, *I*_T_. The data acquisition system, DAQ, records the intensities and transmits the data to a PC (not shown)

The experimental arrangement is shown schematically in Fig. 1. In the present work, the multi-mode laser used was a commercially available Fabry–Perot QCL (Thorlabs QF5300CM1) providing 80 mW of power at 5.3 μm using a commercial driver (ILX-Lightwave LDX 3232) and an in-house-built temperature controller. Scanning was effected using a function generator to control the drive current (TTi TG230 2 MHz). The output radiation was passed through an optical interference filter (Spectrogon NB-5360-078 nm) to remove some of the irregular spectral structure, and the spectrum of the resulting output was measured using an FTIR instrument (Perkin Elmer Spectrum 100 FT-IR). This measurement was made only once as the output spectrum was found to be very reproducible. The laser beam was directed to the FTIR by a “flip mirror” which was retracted after these measurements were taken to allow the beam to propagate to the absorption cell and detectors. Measurement of the other laser parameters important for MUMAS is described below. Nitric oxide was contained in a multi-pass Herriot cell with effective path length of 26 m. Pressure in the cell was monitored using different gauges for low and higher pressures (Leybold vacuum CTR90 and 600A-1000T-R12-H21X-X, respectively) with an uncertainty of 0.2 % at the lower pressures. Incident and transmitted laser intensities were measured using separate photodetectors (Vigo PVM-3 TE-10/MIP DC F-20 and Vigo PVI-2 TE-6/MIP DC F-20, respectively) and digitally recorded (Handyscope HS4). The output of this data acquisition system was stored on a PC computer for subsequent analysis.

## Experimental results and analysis

### MUMAS of NO

**Fig. 2:**
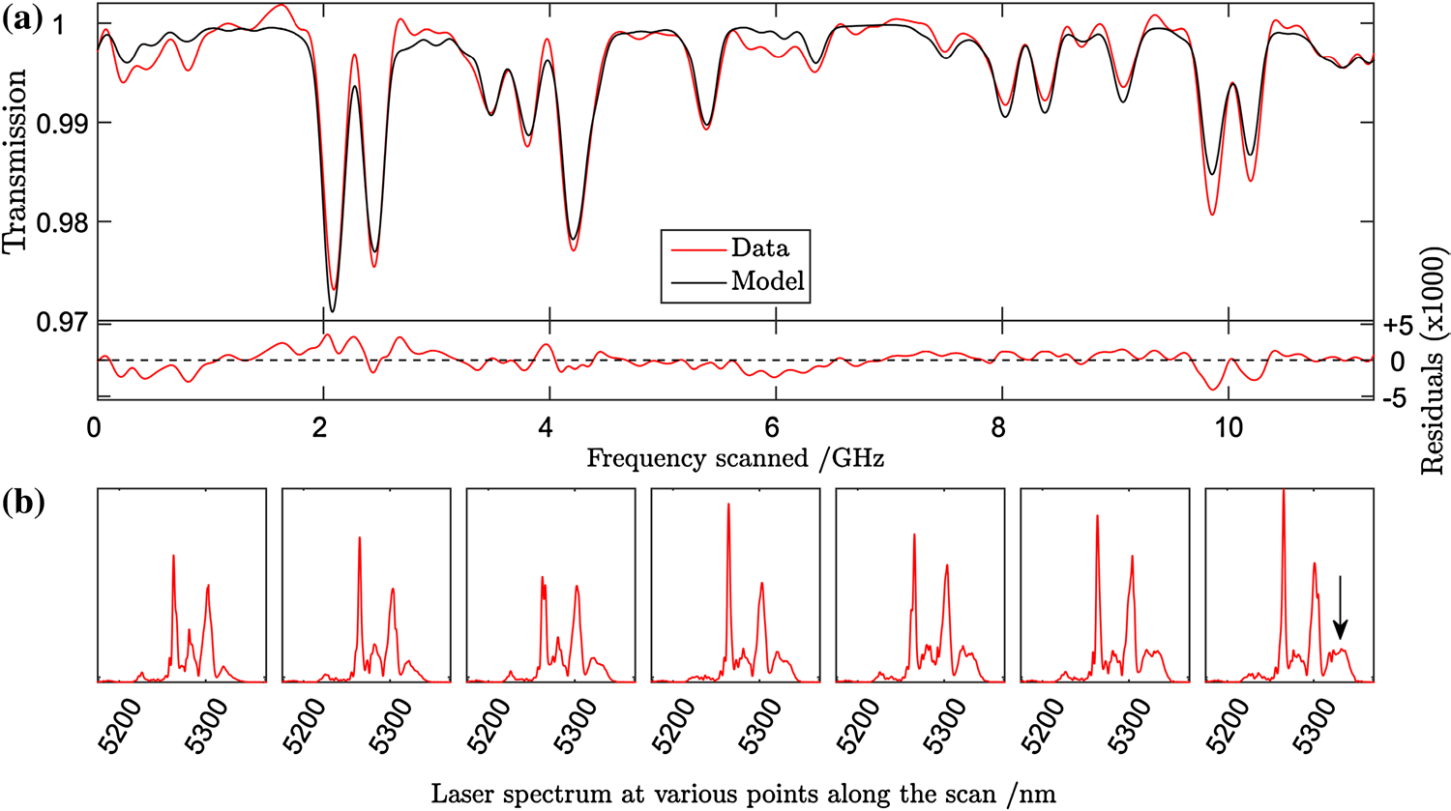
**a** MUMAS spectrum of NO (*red line*) with fitted model spectrum (*black line*). The *lower panel* shows the residual between the data and best-fit model spectrum. **b** Spectral envelopes measured using the FTIR instrument at different locations in the 10 GHz-wide frequency scan of the modes. Note the change in relative intensity of the narrow peaks in the spectrum and the growth of the wide “lobe” beyond 5300 nm towards the end of the scan indicated by the *arrow*

A MUMAS signature of NO, recorded using a pressure of 2.79 mbar is shown in Fig. 2a as the red solid line. These data represent the effect on the total transmitted power as all the modes are scanned across the inter-mode interval, $\Delta \nu_{\text{mode}}$. The absorption features are the result of one or more modes encountering absorption lines of NO during the scan. In a congested spectrum, such as that of NO (which has a rotational constant of similar magnitude to $\Delta \nu_{\text{mode}}$), absorption of several modes, each by a different NO absorption line can contribute to one of the observed “dips” in the transmission. Nonetheless, this MUMAS signature is a unique “fingerprint” corresponding to probing of this species with this particular multi-mode laser. A modelled fit to the data is shown in Fig. 2a as the solid black line. In order to find the best fit to the data the laser parameters, $\Delta \nu_{\text{mode}}$, $\Delta \nu_{{\text{b}{\text{and}}}}$, $\Delta \nu_{\text{width}}$ and $\nu_{\text{m}}$ need to be determined.

As described in previous publications, the location of the modes in frequency space, $\nu_{\text{m}}$, can be estimated using a spectrometer or determined more accurately using cross-correlation with experimental data recorded with a molecule whose absorption lines are well known from a database such as HITRAN [24]. The measurement of the other laser parameters is described below.

### Measurement of mode envelope, $\Delta \nu_{{\text{b}{\text{and}}}}$

Previous MUMAS experiments have used mode envelopes that could be modelled with reasonable accuracy by a simple analytical function [18, 19, 22]. In these cases, the laser provided a smooth and symmetric mode envelope or the output was modified by transmission through an interference filter which also provided a more regular spectral profile. In the present work, the QCL used was a commercially available device which emitted a broadband output with an irregular spectral profile that was not amenable to modelling by a simple analytical function. Some of the spectral intensity variations were removed by passing the laser output through an interference filter (Spectrogon NB-5360-078 nm). This served also to narrow the spectrum to limit the number of transitions probed thus enhancing the relative absorption signals and reducing the number of overlapping transitions recorded. Nonetheless, the resulting spectrum still exhibited significant spectral variation in intensity across the transmitted bandwidth. Consequently, the spectral envelope was measured using a Fourier transform infrared spectrometer, FTIR, for input to the model. The shape of the spectrum, however, was found also to change as the modes were scanned across a single inter-mode spacing, $\Delta \nu_{\text{mode}}$. Figure 2b shows the FTIR measurement of the envelope at seven locations within $\Delta \nu_{\text{mode}}$. For preliminary studies, the envelope was taken to be a constant equal to the average of the spectra measured at these locations and this average function was used to calculate the model spectrum. Errors associated with assuming a fixed spectral profile for the envelope will be discussed below.

### Measurement of inter-mode spacing, $\Delta \nu_{\text{mode}}$

In practice, the most critical parameter for accurate modelling is the inter-mode spacing, $\Delta \nu_{\text{mode}}$, and it is most accurately determined from fits of model signatures to MUMAS data of a known species recorded under known conditions of concentration, temperature and pressure [22, 23]. In the present work, $\Delta \nu_{\text{mode}}$ was determined from fits to MUMAS signatures of NO recorded at low pressure, 2.79 mbar, to maximise spectral resolution. In this way, $\Delta \nu_{\text{mode}}$ was found to be 11.29(5) GHz.

### Measurement of mode linewidth, $\Delta \nu_{\text{width}}$

In some previous demonstrations of MUMAS, the mode linewidth, of the order of several MHz, was insignificant compared to the Doppler and pressure-broadened absorption lines (of order GHz) and so was not a critical parameter in the model [18, 20]. In the case of a multi-mode interband cascade laser, ICL, $\Delta \nu_{\text{width}}$ was measured using MUMAS signatures of a molecule with widely spaced absorption lines so that a feature could be identified as arising from the interaction of a single individual mode with one spectral line [23]. At the operating wavelength of the device used, HCl provided a suitable, open absorption spectrum. The pressure broadening of an isolated line was measured, and the residual width, found by extrapolating to zero pressure, was assigned to that of the mode responsible [23]. In order to find $\Delta \nu_{\text{width}}$ for the QCL used in the present work, an alternative method is employed since no species with a suitable uncongested spectrum was available at 5.3 μm. This method again uses measurement of the pressure broadening of absorption features in a MUMAS spectrum. It is assumed that the lineshape of the individual modes can be well-approximated by a Lorentzian of fixed width, $\Delta \nu_{\text{width}}$. Pressure broadening of the absorption lines is also taken to give a Lorentzian profile with a pressure-dependent width, $\Delta \nu (p)$. In the usual fitting procedure, values are assigned to both the mode linewidth, $\Delta \nu_{\text{width}}$ and the pressure-broadened width, $\Delta \nu (p)$. However, it is not possible to distinguish, a priori, how much of the resulting width, ∆*ν*_Total_, is due to pressure broadening or to the mode linewidth. The following method is adopted to determine the mode linewidth.

**Fig. 3:**
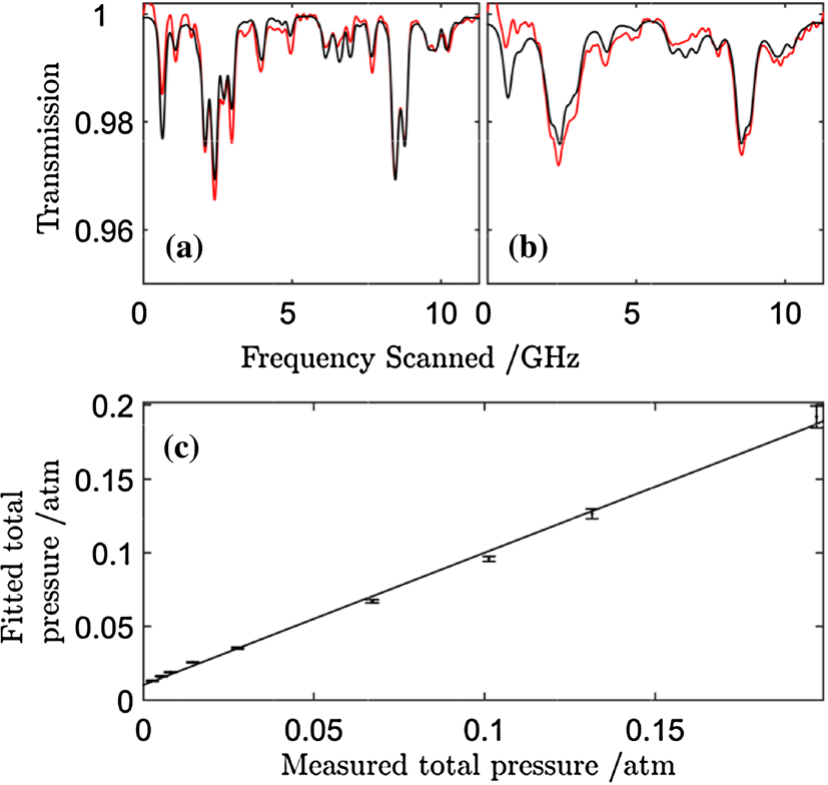
MUMAS spectra of NO at 6.7 mbar with N_2_ at pressures of 0.94 mbar (**a**) and 68 mbar (**b**), respectively. **c** Shows a plot of the pressure derived from fits to the data using pressure-dependent broadening as the fit parameter versus the measured pressure measured

MUMAS spectra were recorded using a partial pressure of 6.7 mbar of NO and variable pressures of N_2_ buffer gas. Modelled MUMAS spectra were fitted to the experimental data in which the mode linewidth was set to zero, i.e. assigning all of the Lorentzian contribution to the linewidth to pressure broadening, ∆*ν*_Total_ = $\Delta \nu (p)$. Figure 3a, b shows examples of MUMAS data recorded with N_2_ pressures of 0.94 mbar and 68 mbar, respectively. The pressure derived from the apparently pressure-broadened linewidths derived from these fits, i.e. the “fitted pressure”, is plotted versus the “measured pressure” and is shown in Fig. 3c. Extrapolating the data to zero measured pressure determines the intercept on the “fitted pressure” axis, i.e. the pressure that would give a Lorentzian broadening equal to the mode linewidth at zero pressure. The data indicate a pressure of 10.3 ± 0.6 mbar. It is then necessary to determine the conversion factor that relates this apparent pressure to the corresponding linewidth.

To find the conversion factor, MUMAS spectra were simulated using a mode linewidth set to zero and a range of buffer gas pressures. For each resulting spectrum, the simulation was repeated but setting the buffer gas pressure to zero and adjusting the mode linewidth to give an equivalent spectrum matching the original. A plot of the equivalent mode linewidth versus the simulated buffer pressure yielded a straight line whose gradient gave the conversion factor of 6.35 mbar MHz^−1^. Thus, the mode linewidth, $\Delta \nu_{\text{width}}$, derived from the fitted pressure intercept at zero measured pressure was found to be 37.62 ± 2.20 MHz. Confirmation of the accuracy of this measurement was obtained by fixing the mode linewidth at this value and deriving the buffer gas pressure from fits to data using the pressure-broadened width as the fit parameter. A plot of derived pressure versus measured pressure yielded a straight line with an intercept of 0.06 ± 0.66 mbar, i.e. zero within experimental error. The slope of the line is 0.9 rather than 1.0 and this deviation is ascribed, as before, to the presence of residual air in the gas-handling system that is not detected by the MUMAS absorption [23].

### Simultaneous NO and H_2_O detection

Water vapour is a common “interfering” species in optical gas sensing owing to its presence in the atmosphere and having absorption lines across a wide range of the infrared region including the region of the NO spectrum studied here. To examine its effect, a MUMAS spectrum was first recorded for pure NO at a pressure of 6.7 mbar in the absorption cell. Then a small amount, 13.3 mbar, of laboratory air was admitted and a second MUMAS spectrum recorded. The laboratory air was measured, using a hygrometer, to have a relative humidity of 24.6 % and a temperature of 26 °C thus providing a water partial pressure of 0.11 mbar in the cell.

**Fig. 4:**
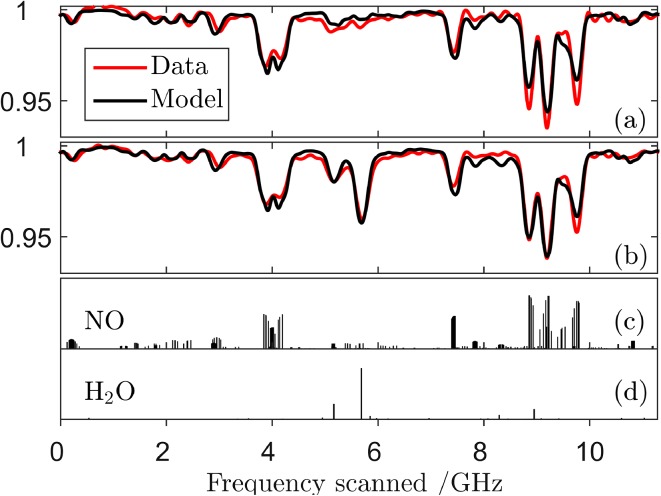
(*a*) MUMAS spectrum of NO at 6.7 mbar (*red line*) with model fit (*black line*) using a constant mode envelope calculated from the average of all spectra recorded at seven locations in the scan shown in Fig. 2b. (*b*) MUMAS spectrum of NO as in (*a*) but with 13.3 mbar of laboratory air added which includes 0.11 mbar partial pressure of water vapour. (*c*), (*d*) *Markers* indicating the position of NO and H_2_O absorption lines, respectively, where they occur during the scan over the inter-mode interval $\Delta \nu_{\text{mode}}$ of about 11 GHz

A MUMAS spectrum of pure NO is shown in Fig. 4(*a*) together with a modelled fit using the laser parameters derived above. The spectrum recorded in the presence of the laboratory air is shown in Fig. 4(*b*) where some of the additional features arising from H_2_O are clearly distinguishable. The lower panels of Fig. 4 show the position of the NO and H_2_O absorption lines, (c) and (d), respectively, where they occur in the scan across the inter-mode interval. This figure therefore does not represent the spectrum of NO or of H_2_O as would be displayed conventionally as a function of wavelength or frequency. Instead, this figure shows a “stick” feature corresponding to a particular transition where it would occur in the scan across $\Delta \nu_{\text{mode}}$ as one of the longitudinal modes encounters it.

These data illustrate the potential for MUMAS to distinguish background “interference” from water, or other species, since additional information on the target species being detected is provided at locations where overlapping of absorption lines is less severe or absent. This information allows the relative contributions of a target species, such as NO, to be distinguished from those of H_2_O.

### Mode envelope effects on data analysis

The data and model-fits shown in Figs. 2 and 4 show reasonable agreement but closer examination reveals some discrepancies, mainly in the relative intensities of absorption features at different positions within the scan across the inter-mode interval, $\Delta \nu_{\text{mode}}$. These slight discrepancies are accounted for by the variation in the shape of the mode envelope as the laser modes are scanned across $\Delta \nu_{\text{mode}}$ as shown in Fig. 2b. As noted above, the analysis of the data and model fitting assumed a fixed (average) mode envelope and so the mode proportional power of each mode in the incident light is assumed to remain constant during the scan.

However, the mode proportional power of the modes actually varies as the distribution of intensity across the modes changes during the scan. Consequently, the contribution of each mode to the total MUMAS signal will depend on how much its proportional power differs from the assumed (average) value when it encounters an absorption line. In principle, account may be taken of the variations by using FTIR measurements of the mode intensity envelope, at each position in the scan. In practice, however, such measurements are affected by the finite resolution of the instrument and so it is necessary to measure the instrument function of the FTIR interferometer. We note that the variation in the mode intensity envelope is correlated with the modulation of the drive current and is not randomly varying. The reproducibility of these variations allows them to be accounted for in the model and in fitting to the data.

Inspection of Fig. 2b shows that, in particular, the spectrum develops a broad “lobe” at the long-wavelength end of the distribution as the QCL emission modes are scanned across $\Delta \nu_{\text{mode}}$. The overall output power changes also, but the important issue is that the proportion of the total power contained in any individual mode will change as this lobe develops. The mode proportional power of a mode can increase or decrease during the scan and so its contribution to the total MUMAS signal could increase or decrease correspondingly relative to that predicted for the assumed average value of the envelope. It is possible, in principle, to calculate the contribution of each mode as its mode proportional power changes during the scan. However, this would be computationally expensive and require accurate, high resolution, measurements monitoring the continuous variation of the envelope during a scan. The range of uncertainty introduced by variation of mode proportional power may be estimated by calculation of the fitted modelled MUMAS spectra that would arise if the mode proportional power varied between the maximum and minimum possible values. Alternatively, the fit may be adjusted at particular locations in the scan to take account of significant changes in the mode proportional power since the effect of such changes will depend on the strength of any known absorption features occurring at that point.

### Measurement of relative mode intensities

**Fig. 5:**
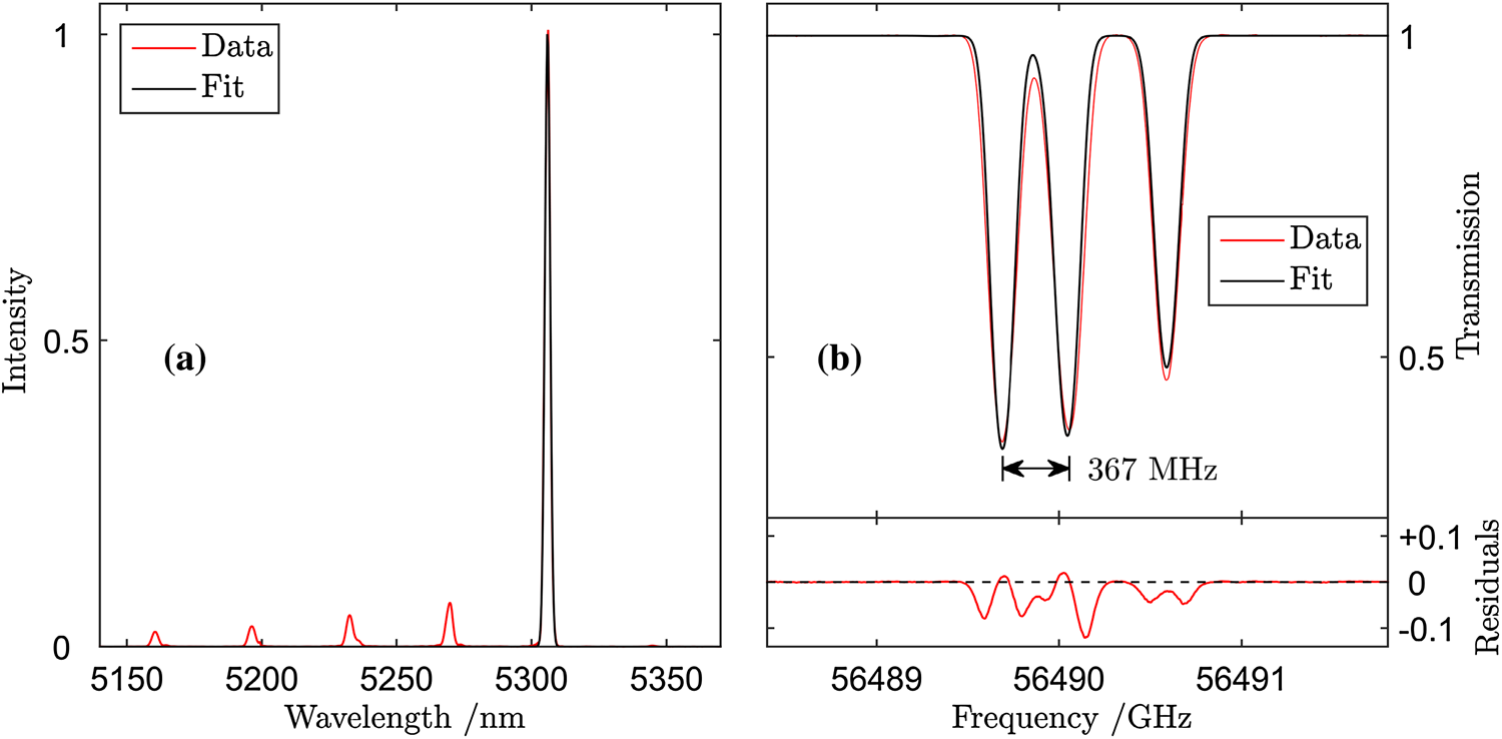
**a** FTIR spectrum of QCL output at high drive current resulting in output dominated by a single mode. The *black line* indicates a Gaussian fit to the dominant single mode and represents the instrument function. **b** MUMAS spectra of the *R*(1.5) *v* = 1 < −0 ro-vibrational transitions in NO recorded using the QCL operating in a single longitudinal mode, data is shown as *red line* and the modelled fit as *black line*

As noted above, measurement of the spectral distribution of intensity across the mode envelope needs to account for the finite resolution of the FTIR instrument. The recorded FTIR intensity spectrum of the mode envelope is a convolution of the actual mode spectrum with the instrument function. Thus, the relative intensities of individual modes can be recovered fully only if the instrument function is de-convolved from the measured data. The instrument function of the FTIR interferometer was measured by recording the spectrum of a monochromatic source. Such an effectively monochromatic source, i.e. having a linewidth very much smaller than the instrument width, was produced by inducing the QCL used for the MUMAS experiments to operate in a single longitudinal mode by increasing the drive current. Figure 5a shows the FTIR spectrum of the laser output under these operating conditions and shows it to be dominated by a single mode at around 5300 nm. To gain an estimation of the mode linewidth, the laser was used to record an absorption spectrum of both Λ-doublet components of the *R*(1.5) *v* = 1 < −0 ro-vibrational transitions within the Ω = 3/2 and 1/2 spin–orbit manifolds of NO. The spectrum shown in Fig. 5b was obtained using 2.63 mbar of NO in the Herriot cell and scanning the laser over 6 GHz. Also shown is a modelled spectrum fitted to the experimental data. The relatively large residual is ascribed to the uncertainty arising from the frequency calibration of the spectrum using fringes formed by a Germanium etalon. Since the laser was scanned across this relatively narrow spectral range, there is a relatively large uncertainty in the frequency scale. Nonetheless, the main point of this figure is to show the high spectral resolution provided by the laser operating in a single mode. The spectrum consists of well-resolved Doppler limited features in which the splitting of the *e*/*f* Λ-doublet components within the Ω = 1/2 manifold of 367 MHz is readily resolved, indicating the width of the mode has an upper limit of about 10 MHz and considerably narrower than the nominal 0.5 cm^−1^ (15 GHz) resolution of the FTIR spectrometer. As expected the splitting between the *e*/*f* Λ-doublet components of the Ω = 3/2 manifold is too small to be resolved.

This data shows that, even if some additional longitudinal modes are activated, the vast majority of the output under these drive conditions resides in a single mode as the spectrum simulated, assuming a single mode, accounts for practically all of the measured absorption. Additional confirmation of single-mode operation was found using a self-heterodyne measurement that indicated a mode linewidth of less than or equal to 1 MHz [25]. We note that when the laser is operated multi-mode with lower drive current, and lower gain, the mode linewidth is significantly increased to a value of the order of 37 MHz as described above.

**Fig. 6:**
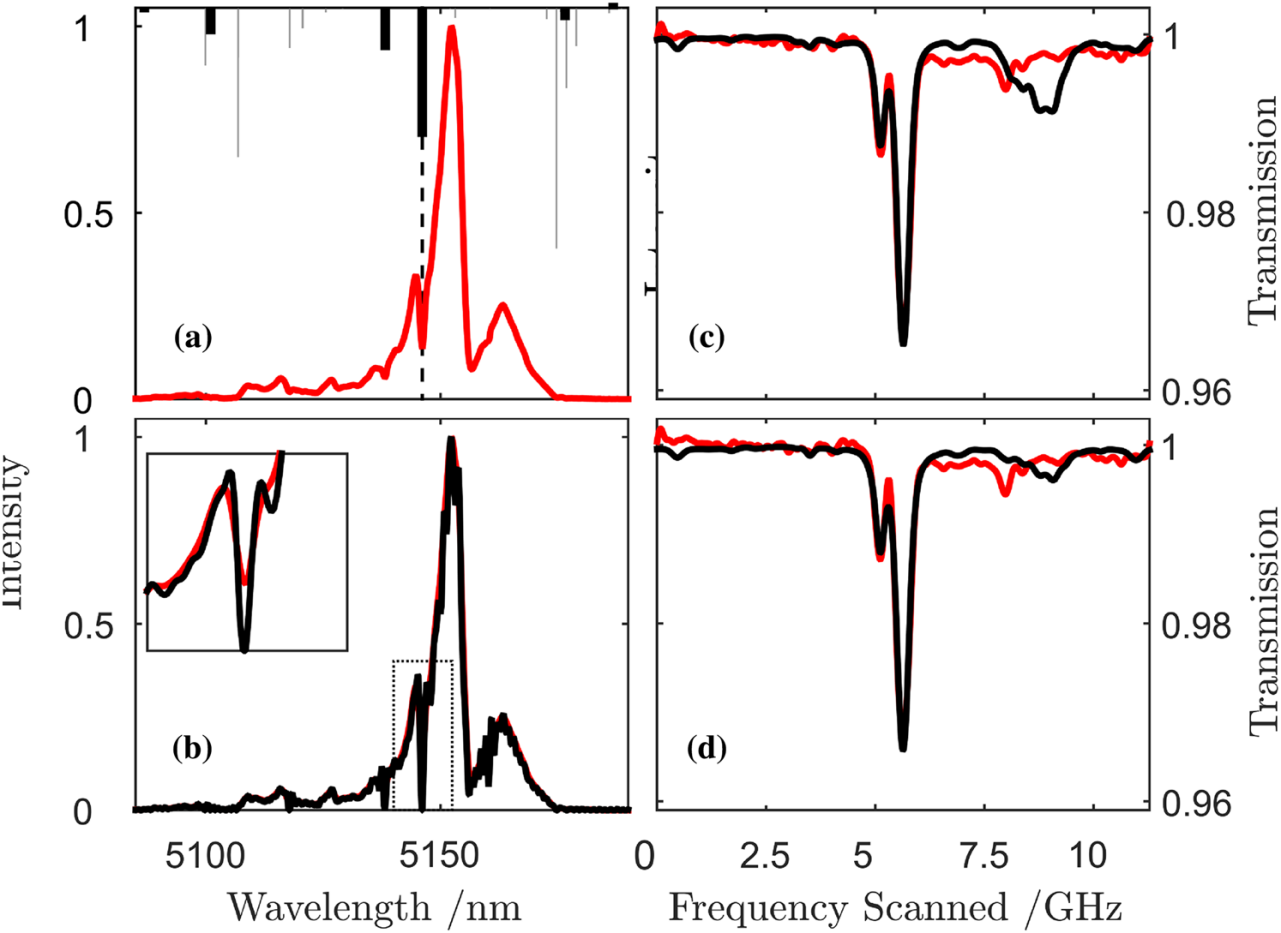
**a** FTIR spectrum of QCL laser output with stick spectra (*upper plot*) indicating positions of *spectral lines* of H_2_O in this region. Note the strong feature lying at the location of the pronounced dip in the spectrum near 5150 nm. **b** The output spectrum when the instrument function is de-convolved from the *data* in (**a**). Note that the dip around 5150 nm reaches zero intensity whereas the unresolved spectrum in **a** indicates a finite intensity at this spectral location. **c** MUMAS spectrum of H_2_O (*red line*) with model fit (*black line*) showing over-prediction of contribution of the transition highlighted in **a** around 8 GHz in the scan. **d** Same MUMAS data as shown in **c** but with model re-calculated using the de-convolved FTIR spectrum of **b** showing reduction in the feature at 8 GHz in better agreement with the data

The measured instrument function at 5.3 μm, shown in Fig. 5a, was found to be well-fitted by a Gaussian function. Using this analytical function, the FTIR spectra recorded at each of the different locations in the scan across the inter-mode spacing as shown in Fig. 2b were de-convolved to yield a higher resolution representation of the spectral shape of the mode envelope. Such a FTIR spectrum of the QCL operating multi-mode is shown in Fig. 6a as the red line. The de-convolved spectrum is shown as the black line in Fig. 6b and exhibits several sharp dips where the actual mode intensity is effectively zero. This is in contrast to an assumed non-zero value based on the unresolved average spectrum (See inset to Fig. 6b).

### Effects of changing mode proportional power on MUMAS spectra

A MUMAS spectrum of H_2_O present in a sample of laboratory air at 0.11 mbar pressure is shown in Fig. 6c as the red line. A “best fit” modelled MUMAS spectrum is indicated in the figure as a black line which has been calculated using the mode envelope derived using the averaged FTIR spectrum without de-convolution, i.e. the unresolved average spectrum. The modelled MUMAS spectrum shows some features between 8 and 9 GHz in the scan that do not appear in the experimental data. In Fig. 6a, several water transitions lying within the un-resolved mode envelope are shown in the upper part of the figure. The dotted line in Fig. 6a indicates the position of a particularly strong transition of H_2_O which coincides with the pronounced dip in the mode envelope. Since the averaged, and unresolved, envelope assumes more mode proportional power in the mode or modes interacting with these absorption lines than is actually the case, the model will predict the features shown between 8 and 9 GHz as seen in Fig. 6c. However, when the de-convolved envelope spectrum is used to construct the model MUMAS spectrum these features are significantly suppressed as shown in Fig. 6d where the model is seen to fit better the data across the whole scan range.

**Fig. 7:**
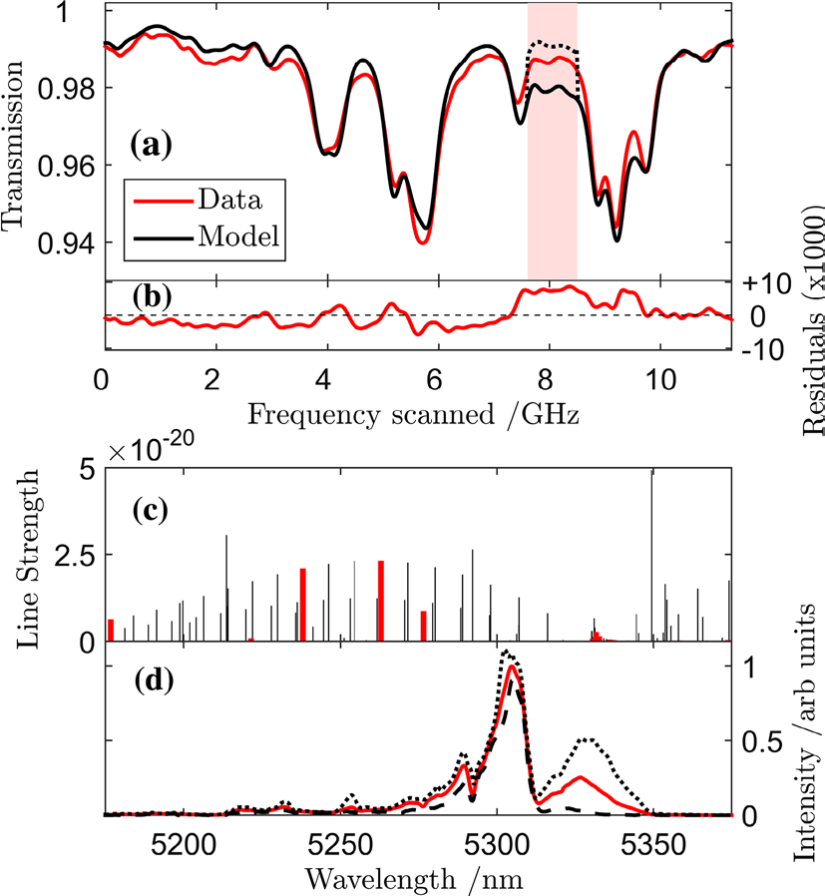
MUMAS spectrum of NO and H_2_O illustrating the effect of changing mode proportional power. **a** *Data* shown as *red line* with a model fit using the averaged mode envelope shown as the continuous *black line*. **b** Residual of fitted spectrum showing increased discrepancy around 8 GHz. **c** Stick spectrum indicating the *lines* in NO and H_2_O contributing to the spectrum in **a** as a function of wavelength. The *line strength* is given in units of cm^−1^/(molecule × cm^−2^). The features marked as *red bold lines* are those responsible for the features at 8 GHz in the scan. **d** FTIR spectrum of QCL output. The *solid red line* is the averaged spectrum over the scan. The *dotted lines* indicate the spectral shape at the beginning and end of the scan showing increasing intensity lobe between 5300 and 5350 nm. The model spectrum calculated using the mode envelope including this lobe results in the *dotted line* shown in **a** around 8 GHz

These effects are apparent also in the MUMAS spectra of the NO and H_2_O. Figure 7a shows an experimental MUMAS spectrum of the mixture (6.7 μbar NO and 0.44 mbar H_2_O) together with a model spectrum fitted to the data using the averaged and unresolved mode envelope. The residual of the best-fit model is shown in Fig. 7b shows a particular discrepancy around 8 GHz in the scan. The transitions contributing to the signal at this point in the scan are indicated by the red lines in Fig. 7c and are mostly due to H_2_O with some contribution from clusters of weaker NO lines. In Fig. 7d, the averaged mode envelope, as measured by the FTIR instrument, is shown as the solid red line. The two envelopes indicated by the dashed and dotted lines show the spectra at the beginning and end of the scan, respectively, from which it can be seen that the lobe on the envelope develops significantly around 5330 nm during the scan. This variation leads to a change in the mode proportional power of those modes responsible for the features at 8 GHz. The model MUMAS spectrum was re-calculated for the part of the scan indicated by the shaded column in Fig. 7a around 8 GHz, using the form of the mode envelope appropriate to this part of the scan. The result is that the over-prediction of MUMAS signal in this region is reduced and the model signal, shown by the dotted line in Fig. 7a, in the region of 8 GHz, is in much better agreement with the data. It is worth noting that, as indicated by their spectral location as seen in Fig. 7c, the lines responsible for the feature at 8 GHz, do not lie in the region where the mode envelope changes most dramatically. The change in their contribution arises from the change in their mode proportional power as the growing lobe at 5330 nm accounts for more of the total power of the laser.

It is also interesting to note that “strong” absorption features are detected even at the low partial pressure of 6.7 μbar. These features arise from the cumulative effect of many “weak” transitions being interrogated by multiple modes such that they occur at approximately the same location in the scan across the inter-mode spacing.

### Rapid scanning MUMAS

**Fig. 8:**
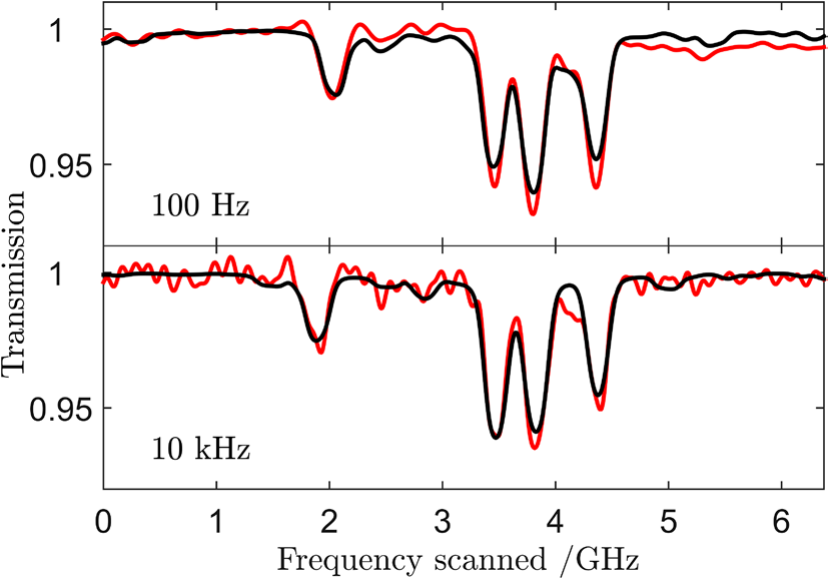
MUMAS of NO at scan rates of 100 Hz and 10 kHz. The features lying between 2 and 3 GHz in the scans show slightly different profiles owing to the slight change in mode spacing arising from a slight change in drive current used

All the MUMAS data shown above were recorded whilst scanning the longitudinal modes at a rate of 100 Hz across the inter-mode interval of approximately 10 GHz. The laser used in these studies is capable of being scanned at significantly higher rates. However, the amplitude of the frequency scan was found to decrease at higher scan rates owing to limitations imposed by the drive electronics available. Figure 8a, b shows data recorded over a range of 6 GHz at scan rates of 100 Hz and 10 kHz, respectively, from which it can be seen that the resolution of the spectra is not significantly reduced at these higher scan rates. At a scan rate of 50 kHz the scan range reduced to only 2 GHz. This limited range is still sufficient to include the cluster of features arising from several absorption lines at different 2 GHz wide sections within the total bandwidth of the laser. It can be seen in Fig. 8a that some features in the data and fitted spectrum between 2 and 3 GHz do not appear at the higher scan rate in Fig. 8b. This difference is accounted for by the slightly different laser drive current used at the higher scan rate which resulted in a slightly changed inter-mode interval. The drive current was adjusted between the two conditions in order to avoid some transient features that appeared at the start of the scan at the higher rate. Thus, some extra absorption features were detected at 100 Hz that are absent at 10 kHz. These results, however, demonstrate the potential for gas sensing with fast response times. Drive electronics with a higher bandwidth should allow an increase in scan rate with consequent decrease in measurement times. In process control applications or studies of rapidly changing molecular concentrations, fast response times (~10 s of μs) will be advantageous.

## Conclusions

This work has demonstrated the first application of a QCL to MUMAS in the mid-infrared region of the spectrum. Multiple transitions in the NO molecule were detected over a bandwidth of 50 cm^−1^ determined by the transmission of the interference filter. In addition, in the presence of laboratory air containing water vapour, transitions in H_2_O were simultaneously recorded, demonstrating the multi-species sensing capability of MUMAS. A particular feature of the work shows that MUMAS can be applied using a commercially available QCL device that exhibits not only strong variations in the spectral intensity distribution but also changes in the mode proportional power during the scan over the inter-mode spacing. It has been shown that including such variations of the QCL output, measured independently, in the modelling procedure allows good fits to experimental data from which partial pressures of the gas species present can be derived. Finally, it has been shown that MUMAS data can be obtained at high scan rates, up to 10 kHz, and beyond if a suitable driver is available, to enable gas sensing with short measurement times important for applications in rapidly changing environments or for active process control.
